# PD1/PD-L1 dependent immunosuppression by huiPS-derived cell population

**DOI:** 10.1186/1479-5876-9-S2-P31

**Published:** 2011-11-23

**Authors:** Jonathan Pini, Arieh Moussaieff, Emilie Murris, Reuven Shalom-Feuerstein, Isabelle Petit, Daniel Aberdam, Matthieu Rouleau

**Affiliations:** 1 U898 INSERM, University of Nice Sophia Antipolis, Nice, France; 2 INSERTECH, Bruce Rappaport Institute of the Technion, Haifa, Israel

## Background

Human induced pluripotent stem cells (huiPS) are produced by *in vitro* reprogrammation of somatic cells. They offer new perspectives in cell therapy due to their ability to differentiate in any cell types. Nevertheless, used either in allotransplantation (different donor and recipient), or in autologous tranplantation (patient’s derived iPS), iPS derived cells can induce rejecting immune responses [1]. It appears, therefore, crucial to better characterize their immunogenicity.

## Methods

Applying to human iPS cells an *in vitro* ectodermal differentiation protocol (cellular matrix and BMP4 treatment)– initially designed in the laboratory for embryonic stem cells [2] - we isolated a cell population that can serve to analyse immunogenicity of iPS-derived cells.

## Results

We demonstrate that these cells are unable to directely activate human lymphocytes, but instead display an immunosuppressive activity on allogenic activated T lymphocytes as shown by a strong inhibition of allogenic CD4- and CD8-positive T cell proliferation and reduction in IL-2 and IFN-gamma production. While immunossupressive soluble factors such as IL-10 or TGF-beta are not involved, we demonstrate, with the use of blocking antibodies, that this immunosuppressive activity rely on the inhibitory interaction between the PD1 receptor (upregulated on T cells) and its ligand PD-1L strongly expressed by our human iPS –derived population.

Finally, we demonstrate that this cell population contains mesenchymal stem cells, as shown by immunophenotyping, cytokines production and *in vitro* differentiation into adipocytes, osteoblasts and chondrocytes.

## Conclusions

These results suggest that during *in vitro* human iPS cell differentiation into defined cell populations with strong interest in cellular therapy, some mesenchymal stem cells with immunosuppressive activity may differentiate and limit the immune process suppose to induce their rejection. Interestingly, the mechanisms involved might *in fine* participate in establishing a long term immune tolerance.
